# Comparative Efficacy and Safety of Low-Dose Versus Standard-Dose Rabbit Antithymocyte Globulin Induction Strategy in Kidney Transplant Recipients: Insights From a Single-Center Experience in North India

**DOI:** 10.7759/cureus.69770

**Published:** 2024-09-20

**Authors:** Dinesh Khullar, Deepak Kumar Panigrahi, Sahil Bagai, Abhishek, Kulwant Singh, Kunal Raj Gandhi, Pallavi Prasad, Rahul Grover, Gagandeep Chhabra, Narinder Pal Singh, Anish Kumar Gupta

**Affiliations:** 1 Nephrology, Max Super Speciality Hospital, Saket, New Delhi, IND; 2 Department of Nephrology, Amrita Hospitals, Faridabad, IND; 3 Nephrology, Vardhman Mahavir Medical College and Safdarjung Hospital, New Delhi, IND

**Keywords:** antithymocyte globulin, immunosuppressant therapy, immunosuppression, induction therapy, kidney transplantation

## Abstract

Background

Rabbit antithymocyte globulin (rATG) is frequently utilized as an induction therapy in kidney transplant recipients (KTRs). Full-dose rATG induction therapy (7-10 mg/kg) has been associated with increased morbidity. However, definitive data on the appropriate rATG dosage remains scarce. In this study, we evaluated the efficacy and tolerability of varying rATG doses in KTRs.

Methodology

A single-center, retrospective, observational study was conducted between 2009 and 2014 in a cohort of 208 KTRs who received rATG induction therapy. Patients included in the study had received two to three consecutive doses of rATG as part of their planned induction protocol. Participants were categorized into the following two groups based on the cumulative dosage of rATG received during induction therapy: group A received 2 or 2.5 mg/kg, while group B received ≥3 mg/kg. The five-year follow-up data were analyzed.

Results

A cumulative rATG dose of 2 or 2.5 mg/kg and ≥3 mg/kg was given to 122 and 86 patients, respectively. The incidence of delayed graft function (DGF), acute rejection episodes, total graft loss, death, and death-censored graft loss was 6.25%, 3.84%, 7.21%, 4.32%, and 2.88%, respectively. Two malignancies and 141 infectious complications were noted. There was no significant difference between the groups regarding DGF, total graft loss, death, death-censored graft loss, infectious complications, and incidence of acute rejection episodes. Deceased donor kidney transplantation was identified as a significant predictor of acute rejection episodes (odds ratio = 9.19, 95% confidence interval = 1.567-53.907; p = 0.014).

Conclusions

The dosage for rATG induction therapy for KTRs should be tailored based on immunological and other factors impacting graft survival, along with a comprehensive risk assessment for potential infectious complications. A cumulative dose of 2.0 or 2.5 mg/kg could be optimal, offering effective induction therapy in KTRs with excellent graft survival rates and potentially fewer infectious complications.

## Introduction

Kidney transplantation is the most effective treatment option for end-stage kidney disease (ESKD). Kidney transplant recipients (KTRs) need potent immunosuppressive therapy to prevent rejection. Antithymocyte globulin (ATG) is used both as induction therapy and to treat acute rejection in organ transplantation [1,2]. Induction therapy with ATG has been shown to decrease the rate of acute rejection episodes along with the possibility of developing infections and malignancies [3-8]. The first clinical study on rabbit antithymocyte globulin (rATG) as induction immunotherapy was published in 1971 [9]. Progress continued at a slow pace throughout the 1990s until the first randomized double-blind comparison of rATG and equine ATG for induction therapy was reported in 1999 [10-12]. The Kidney Disease Improving Global Outcomes (KDIGO) guidelines recommend rATG for induction therapy in recipients at high immunologic risk of acute allograft rejection [13]. However, the appropriate induction therapy dosage based on patient risk profiles remains undefined. Several studies have suggested a total dose of 6.0 to 11.5 mg/kg [14,15], but only a few have assessed the effectiveness of lower rATG doses [16,17]. Concerns about the potential adverse effects of rATG, such as pancytopenia, prolonged lymphopenia, infection, and serum sickness, have been raised [18]. Additionally, it has been proposed that lowering the dose could reduce overall costs and mitigate the risks associated with excessive immunosuppression. Further, it has also been postulated that a reduction in the dose may, in turn, reduce the overall financial burden and possibly curtail the risks linked with too much immunosuppression. In the Indian subcontinent, the usual cumulative dose of rATG used is 3 mg/kg. However, at times, it needs to be modified as per the clinical situation. Here, we report our experience with a cumulative rATG dose of 2 or 2.5 mg/kg and ≥3 mg/kg that was given to KTRs as part of their induction immunosuppressive protocol.

## Materials and methods

Study population

This single-center, retrospective, cohort study included patients who underwent kidney transplantation with rATG induction therapy between 2009 and 2014, with follow-up data available for five years. KTRs who received two to three consecutive doses of rATG as part of their planned induction protocol were included in the study. KTRs under 10 years of age, ABO-incompatible transplants, dual-organ transplants, and those with incomplete follow-up data were excluded. Highly sensitized transplants with a positive cross-match status were also excluded.

Study procedure

The primary objective was to study the incidence of acute rejection episodes and the secondary objectives were to study the incidence of delayed graft function (DGF), graft loss, death, and various infectious complications. All necessary clinical and biochemical data were collected retrospectively from hospital records. The cumulative dose of rATG was decided as per the treating nephrologist’s decision at that point in time. The rATG dose was administered with appropriate dilution in normal saline following premedication with steroids and antihistamines intraoperatively, with subsequent daily doses. Dosing was deferred if the total leucocyte count was less than 3,000/µL. Preoperatively, tacrolimus (0.1 mg/kg) was initiated, targeting trough levels of 8-12 ng/dL. Mycophenolate mofetil (MMF) (1,000 mg twice daily) or enteric-coated mycophenolic acid (720 mg twice daily) was also started preoperatively. Injectable methylprednisolone 500 mg was given to all KTRs on the day of surgery, followed by oral prednisolone starting at 0.5 mg/kg/day. This dose was tapered to 15-20 mg at one month and 5 mg per day by the end of the third month post-transplant. All patients were maintained on triple-agent immunosuppression, including prednisolone, a calcineurin inhibitor (tacrolimus or cyclosporine), and mycophenolic acid (MMF or enteric-coated mycophenolate sodium).

Recipients were followed up twice a week for the first month, once a week for the next three months, once a month for the next six months, and then every two months thereafter. Universal cytomegalovirus prophylaxis with oral valganciclovir was provided for three months, and cotrimoxazole prophylaxis was given for 12 months to all patients. Infections were treated with appropriate antibiotics based on local antibiograms and culture reports. DGF was defined as a complication needing hemodialysis in the first week post-transplant. Acute cellular rejections were treated with methylprednisolone pulses, while antibody-mediated rejections were managed with plasmapheresis and intravenous immunoglobulins. Graft loss was defined as an estimated glomerular filtration rate of less than 15 mL/minute/1.73 m^2^.

Ethical considerations

The study was approved by the ethics committee (EC) of Max Healthcare, New Delhi (EC approval number: BHR/RS/MSSH/MHIL/SKT-1/MHEC/NEPHRO/24-06). The informed consent requirement was waived as the research was conducted on anonymized patient data. The study adhered to the ethical principles of the Helsinki Declaration and followed the applicable guidelines for Good Clinical Practice.

Statistical analysis

Data were collected retrospectively at discharge, six months, one year, three years, and five years post-transplant. The outcomes evaluated included DGF, acute rejection episodes, total graft loss, death, death-censored graft loss, malignancy, and infectious complications. SPSS version 22.0 (IBM Corp., Armonk, NY, USA) was used for data analysis. Results were reported as mean (standard deviation), median (interquartile range), and frequency (percentage). Categorical variables were compared using the chi-square test or Fisher’s exact test, as appropriate. Continuous variables were compared using the independent t-test for normally distributed data or the Wilcoxon rank-sum test for non-normally distributed data. Univariate logistic regression was utilized for predictors of acute rejections. A p-value of less than 0.05 was considered significant.

## Results

Baseline characteristics of recipients and donors

A total of 208 KTRs were enrolled according to the inclusion criteria. The baseline characteristics of both recipients and donors are detailed in Table 1. The average age of the recipients was 41.65 ± 12.14 years. Among them, 162 (77.88%) were male, and the majority (95.67%) received kidneys from living donors.

**Table 1:** Study participants’ demographic characteristics and clinical outcomes. Group A: rATG dose 2/2.5 mg/kg; Group B: rATG dose ≥3 mg/kg; CGN: chronic glomerulonephritis; CTID: chronic tubulointerstitial disease; DN: diabetic nephropathy; ADPKD: autosomal dominant polycystic kidney disease; IQR: interquartile range; rATG: rabbit antithymocyte globulin; DGF: delayed graft function; NODAT: new-onset diabetes after transplantation; UTI: urinary tract infection; LRTI: lower respiratory tract infection; TB: tuberculosis; CMV: cytomegalovirus; BKV: BK virus

Parameters	Group A (n = 122)	Group B (n = 86)	P-value
Recipient			
Age, years, mean ± SD	41.31 ± 12.23	42.13 ± 12.08	0.634
Gender, N (%), males	98 (80.32)	64 (74.42)	0.312
Weight, kg, mean ± SD	64.13 ± 15.62	61.43 ± 12.63	0.188
Basic kidney disease, N (%)			
CGN	3 (2.45)	2 (2.33)	0.933
CTID	9 (7.37)	6 (6.98)	
DN	30 (24.59)	22 (25.58)	
Presumed CGN	60 (49.18)	37 (43.02)	
ADPKD	1 (0.81)	1 (1.16)	
Unclassified	19 (15.57)	18 (20.93)	
Re-transplantation, N (%)	2 (1.63)	1 (1.16)	0.777
Dialysis vintage, years, median (IQR)	4 (2–8)	4 (2.5–6)	0.229
Blood group, N (%)			
A	32 (26.22)	16 (18.60)	0.180
AB	11 (9.01)	7 (8.14)	
B	55 (45.08)	35 (40.70)	
O	24 (19.67)	28 (32.56)	
Donor			
Age, years, mean ± SD	48.36 ± 10.97	45.17 ± 10.34	0.034
Gender, N (%), females	89 (72.95)	62 (72.09)	0.891
Biological Relationship, N (%), unrelated	76 (62.29)	64 (74.42)	0.066
Deceased Donor, N (%)	7 (5.73)	2 (2.32)	0.234
Number of HLA mismatch, mean ± SD	3.55 ± 1.06	4.13 ± 1.04	0.000
Immunosuppressant			
Mean rATG dose in mg/kg, mean ± SD	2.18 ± 0.24	3.14 ± 0.35	0.000
Cyclosporine use, N (%)	12 (9.8)	5 (5.8)	0.297
mTOR inhibitor use, N (%)	9 (7.4)	12 (14)	0.121
Tacrolimus level, mean ± SD			
At discharge	10.63 ± 2.69	10.58 ± 3.03	0.896
At 6th month	7.02 ± 1.43	7.04 ± 1.80	0.917
At 1st year	6.18 ± 1.36	6.19 ± 1.46	0.968
At 3rd year	5.47 ± 1.15	5.89 ± 1.24	0.019
At 5th year	5.47 ± 0.96	5.51 ± 0.91	0.792
Clinical outcomes			
Serum creatinine, mean ± SD			
At discharge	1.22 ± 0.69	1.20 ± 0.86	0.846
At 6th month	1.35 ± 0.67	1.26 ± 0.37	0.256
At 1st year	1.27 ± 0.36	1.23 ± 0.36	0.514
At 3rd year	1.38 ± 0.67	1.40 ± 1.00	0.869
At 5th year	1.52 ± 0.92	1.38 ± 0.65	0.255
DGF, N (%)	9 (7.37)	4 (4.65)	0.424
NODAT, N (%)	26 (21.31)	22 (25.58)	0.472
Bacterial UTI, N (%)	33 (27.04)	24 (27.90)	0.891
Bacterial LRTI, N (%)	12 (9.83)	3 (3.48)	0.081
TB, N (%)	6 (4.91)	4 (4.65)	0.929
Other bacterial infections, N (%)	7 (5.73)	10 (11.62)	0.127
Fungal infections, N (%)	3 (2.45)	2 (2.32)	0.951
CMV disease, N (%)	5 (4.09)	5 (5.81)	0.569
Leucopenia N (%)	46 (37.7)	35 (40.6)	0.240
BKV nephropathy, N (%)	1 (0.81)	2 (2.32)	0.370
Malignancy, N (%)	2 (1.63)	0	-
Acute rejection episodes, N (%)	5 (4.09)	3 (3.49)	0.822
Total graft loss, N (%)	9 (7.37)	6 (6.97)	0.912
Death, N (%)	5 (4.09)	4 (4.65)	0.847
Death-censored graft loss, N (%)	4 (3.27)	2 (2.32)	0.686

rATG dosing and administration

The rATG dose was determined based on the actual body weight of the patients at the time of their transplant. A total cumulative rATG dose of either 2 or 2.5 mg/kg or ≥3 mg/kg was administered to 122 (58.65%) patients and 86 (41.35%) patients, respectively. They were designated as groups A and B, respectively. The average dose received was 2.18 ± 0.24 mg/kg for group A and 3.14 ± 0.36 mg/kg for group B.

The overall incidence of DGF was 6.25%, with no significant statistical difference in DGF rates between the two rATG dose groups. There were eight (3.84%) episodes of biopsy-proven acute rejection (BPAR) observed across the study population, and the incidence of BPAR did not differ significantly between groups A and B (p = 0.822). A total of 15 (7.21%) KTRs experienced graft loss, but the incidence of total graft loss showed no statistical difference between the two dose groups. Additionally, nine (4.32%) KTRs died during the follow-up period, and death-censored graft loss occurred in six (2.88%) cases, with no significant differences observed between the two rATG dose groups regarding these outcomes. The timeline of these events is detailed in Table 2.

**Table 2:** Post-transplant outcomes with respect to the time period. Group A: rATG dose 2/2.5 mg/kg; Group B: rATG dose ≥3 mg/kg

Endpoint	Total (N = 208)	Group A (n = 122)	Group B (n = 86)
Acute rejection episodes, n (%)	8 (3.84)	5 (4.09)	3 (3.49)
Immediate postoperative period	0 (0.0)	0 (0.0)	0 (0.0)
From discharge up to the end of the 6th month	3 (1.44)	1 (0.82)	2 (2.33)
From the 7th month up to the end of the 1st year	2 (0.96)	1 (0.82)	1 (1.16)
From the 2nd year up to the end of the 5th year	3 (1.44)	3 (2.46)	0 (0.0)
Total graft loss, n (%)	15 (7.21)	9 (7.37)	6 (6.98)
Immediate postoperative period	1 (0.48)	0(0.0)	1 (1.16)
From discharge up to the end of the 6th month	3 (1.44)	2 (1.64)	1 (1.16)
From the 7th month up to the end of the 1st year	3 (1.44)	2 (1.64)	1 (1.16)
From the 2nd year up to the end of the 5th year	8 (3.84)	5 (4.10)	3 (3.49)
Death, n (%)	9 (4.32)	5 (4.09)	4 (4.65)
Immediate postoperative period	1 (0.48)	0 (0.0)	1 (1.16)
From discharge up to the end of the 6th month	2 (0.96)	1 (0.82)	1 (1.16)
From the 7th month up to the end of the 1st year	3 (1.44)	2 (1.63)	1 (1.16)
From the 2nd year up to the end of the 5th year	3 (1.44)	2 (1.63)	1 (1.16)
Death-censored graft loss, n (%)	6 (2.88)	4 (3.27)	2 (2.33)
Immediate postoperative period	0 (0.0)	0 (0.0)	0 (0.0)
From discharge up to the end of the 6th month	1 (0.48)	1 (0.82)	0 (0.0)
From the 7th month up to the end of the 1st year	0 (0.0)	0 (0.0)	0 (0.0)
From the 2nd year up to the end of the 5th year	5 (2.40)	3 (2.46)	2 (2.33)

Malignancy and infectious complications

**Figure 1:**
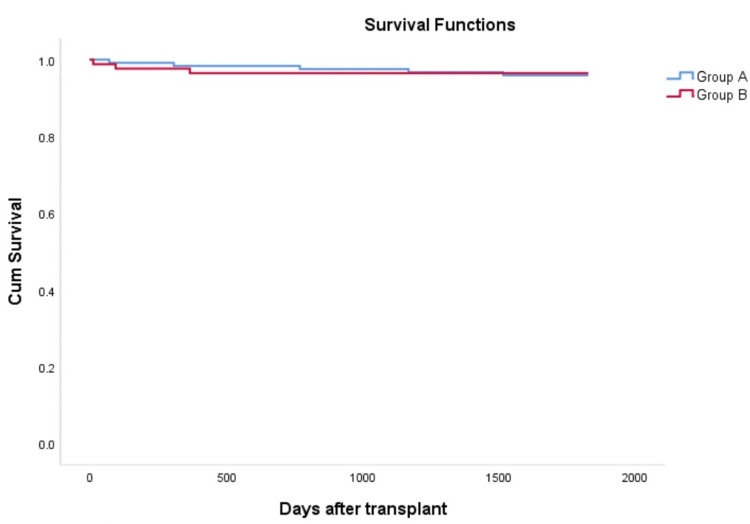
Kaplan-Meier survival estimates for acute rejection-free survival. Log-rank statistic = 0.044, p-value = 0.834.

A total of 141 infectious complications were reported at various time points (Table 3). Bacterial infections were the most common, accounting for 122 (86.52%) episodes, with urinary tract infections (UTIs) being the most frequently encountered type. During the study period, two (0.96%) individuals developed malignancies. The Kaplan-Meier survival analysis indicated no statistically significant difference in rejection-free survival between the two groups (p = 0.834), as shown in Figure 1.

**Table 3:** Infectious complications with respect to the time period. Group A: rATG dose 2/2.5 mg/kg; Group B: rATG dose ≥3 mg/kg; CMV: cytomegalovirus; BKV: BK virus

Time point	Total (N = 208)	Group A (n = 122)	Group B (n = 86)
Immediate postoperative period			
Bacterial infection	37 (17.78)	19 (15.57)	18 (20.93)
Fungal infection	2 (0.96)	2 (1.64)	0 (0.0)
CMV infection	0 (0.0)	0 (0.0)	0 (0.0)
BKV infection	0 (0.0)	0 (0.0)	0 (0.0)
From discharge up to the end of the 6th month			
Bacterial infection	33 (15.86)	21 (17.21)	12 (13.96)
Fungal infection	1 (0.48)	1 (0.82)	0 (0.0)
CMV infection	8 (3.84)	3 (2.46)	5 (5.81)
BKV infection	2 (0.96)	0 (0.0)	2 (2.33)
From the 7th month up to the end of the 1st year			
Bacterial infection	20 (9.61)	9 (7.38)	11 (12.79)
Fungal infection	0 (0.0)	0 (0.0)	0 (0.0)
CMV infection	1 (0.48)	1 (0.82)	0 (0.0)
BKV infection	0 (0.0)	0 (0.0)	0 (0.0)
From the 2nd year up to the end of the 5th year			
Bacterial infection	32 (15.38)	19 (15.57)	13 (15.12)
Fungal infection	2 (0.96)	0 (0.0)	2 (2.33)
CMV infection	2 (0.96)	2 (1.64)	0 (0.0)
BKV infection	1 (0.48)	1 (0.82)	0 (0.0)

KTRs who experienced BPAR had a higher incidence of bacterial UTIs, fungal infections, and BK virus-associated nephropathy compared to those without rejection episodes (p < 0.05). The cohort with BPAR also had a significantly higher rate of death-censored graft loss (p < 0.05) (Table 4). Univariate logistic regression analysis revealed that receiving a kidney from a deceased donor was a significant predictor of BPAR in the KTR population (Table 5).

**Table 4:** Comparison between kidney transplant recipients with and without biopsy-proven acute rejection. BPAR: biopsy-proven acute rejection; KTR: kidney transplant recipients; DGF: delayed graft function; UTI: urinary tract infection; LRTI: lower respiratory tract infection; TB: tuberculosis; CMV: cytomegalovirus; BKV: BK virus; ATG: antithymocyte globulin

Parameters	KTR without BPAR (n = 200	KTR with BPAR (n = 8	P-value
Recipient sex, male, N (%)	155 (77.50)	7 (87.50)	0.504
Recipient age, mean ± SD	41.58 ± 12.14	43.50 ± 12.91	0.661
Deceased donor, N (%)	7 (3.50)	2 (25.00)	0.003
Unrelated donor, N (%)	133 (66.50)	07 (87.50)	0.214
ATG dose in mg/kg, mean ± SD	2.58 ± 0.55	2.50 ± 0.46	0.672
DGF, N (%)	12 (6.00)	1 (12.50)	0.456
Bacterial UTI, N (%)	52 (26.00)	5 (62.50)	0.023
Bacterial LRTI, N (%)	15 (7.50)	0 (0.0)	0.421
TB, N (%)	9 (4.50)	1 (12.50)	0.300
Other bacterial infections, N (%)	15 (7.50)	2 (25.00)	0.076
Fungal infections, N (%)	3 (1.50)	2 (25.00)	0.001
CMV infection, N (%)	9 (4.50)	1 (12.50)	0.300
BKV infection, N (%)	2 (1.00)	1 (12.50)	0.007
Any infection, N (%)	79 (39.50)	6 (75.00)	0.045
Total graft loss, N (%)	12 (6.00)	3 (37.50)	0.001
Death-censored graft loss, N (%)	3 (1.50)	3 (37.50)	0.001

**Table 5:** Univariable logistic regression for predictors of acute rejections. DGF: delayed graft function; ATG: antithymocyte globulin; OR: odds ratio; CI: confidence interval

Parameters	OR	95% CI	P-value
Recipient age	1.014	0.955-1.014	0.660
Deceased donor	9.190	1.567-53.907	0.014
Unrelated donor	3.524	0.425-29.254	0.234
Cumulative dose of ATG in mg/kg	0.744	0.191-2.898	0.670
DGF	2.238	0.254-19.701	0.468

## Discussion

The use of rATG has significantly reduced the rate of BPAR in kidney transplantation. Its mechanism of action on immune responses remains somewhat unclear, although it is primarily thought to deplete T cells, with modulation of cell surface antigens also playing a major role [19]. The incidence of BPAR varies across studies. For instance, Brennan et al. reported a 4% incidence of BPAR in the thymoglobulin group with a cumulative dose exceeding 7 mg/kg [12]. Buchler et al. documented a 14% incidence of BPAR in cadaveric donor kidney transplants with an 8.8 mg/kg cumulative dose of rATG [14]. An observational study by Gaber et al. involving 2,322 living donor kidney transplants reported a 93.6% rejection-free survival at five years with an average cumulative dose of 5.29 mg/kg [20]. Gurk-Turner et al. found no difference in BPAR incidence (9.5% vs. 8.8%, p = 0.9) between groups receiving ≤7.5 mg/kg and >7.5 mg/kg of rATG [17]. Wong et al. compared cumulative doses of thymoglobulin (3.0 mg/kg vs. 4.5 mg/kg) and found no significant difference in acute rejection rates or graft function up to 24 months post-transplant [16]. Laftavi et al. demonstrated that a low dose of rATG (3-5 mg/kg) was safe and effective in low-risk KTRs, with no increased rates of malignancy or infections [21]. Sigurjonsdottir et al. studied low-dose rATG in a pediatric cohort, finding BPAR rates of 12.8% and 13.5% in groups receiving ≤3.5 mg/kg and >3.5 mg/kg, respectively, with no significant difference [22].

Our study evaluated the efficacy and safety of different doses of rATG induction therapy in KTR. The incidence of acute rejection was 3.84%, with a 2 or 2.5 mg/kg induction dose proving non-inferior to a ≥3 mg/kg dose in preventing acute rejection (4.09% vs. 3.49%, p = 0.822). This dose was primarily used in the low immunological risk category with a higher number of HLA matches. No significant differences in rejection-free survival, total graft loss, or death-censored graft loss were found between the two groups. In a retrospective study by Singh et al., the one-year rejection rate was 8.3% in non-sensitized living donor KTRs receiving a cumulative rATG dose of 3 mg/kg [23]. Grafals et al. [3] found that a lower ATG dose of 2.25 mg/kg was safe and effective in preventing acute rejection, with no significant differences in graft survival, patient survival, or infections compared to a 3.75 mg/kg dose. There was also a non-significant reduction in leukopenia, CMV, and BK virus infections in the lower-dose group. Schenker et al. documented a 22% BPAR rate in living donor KTRs with a single 1.5 mg/kg rATG dose, suggesting that doses below 2 mg/kg are associated with higher acute rejection rates [24]. A multicentric Indian study by Ray et al. reported a mean rATG dose of 2.6 ± 1.5 mg/kg and a 7.7% incidence of acute rejection at one year [25].

In our cohort, the incidence of DGF was 6.25%, with no significant difference between groups. A multicenter trial found a 31.5% incidence of DGF in high-risk deceased donor kidney recipients receiving thymoglobulin compared to 44.6% with daclizumab [26]. Grafals et al. noted higher DGF incidence in the low-dose rATG group (2.25 mg/kg) compared to the high-dose group (3.75 mg/kg) (40% vs. 14.1%) [3]. Intraoperative rATG administration may help reduce DGF by blocking adhesion molecules involved in ischemia-reperfusion injury [27].

Higher rATG doses can increase the risk of infections and malignancies [28]. In our study, the most common bacterial infection was UTI. Two KTRs developed malignancies, including one case of post-transplant lymphoproliferative disorder over five years. The lower cumulative rATG dose in our study likely contributed to a lower incidence of complications. No significant differences in infection or malignancy rates were found between groups A and B. The incidence of infections was higher in the BPAR cohort, possibly due to higher cumulative immunosuppression.

Regression analysis identified deceased donor kidney transplantation as a significant predictor of acute rejection. Recent studies suggest that while acute rejection rates are similar, long-term graft survival is better in living donor KTRs compared to deceased donor KTRs [29,30]. Other factors such as recipient age, cumulative ATG dose, and DGF were not predictive of acute rejection in our cohort, possibly due to the low BPAR incidence and exclusion of highly sensitized cases, which may have influenced our results.

Despite the promising results, our study has limitations that should be addressed in future research. As a single-center retrospective study, potential biases and unaccounted confounding variables could have influenced our findings. Larger, multicenter studies with diverse populations are needed to confirm these results. Additionally, further research should focus on high-risk KTRs, such as those with high panel reactive antibodies or anti-HLA antibodies, or ABO-incompatible transplants to determine the appropriate rATG dosing in these populations. Longer follow-up periods are also essential to assess the long-term impact of rATG dosing on graft and patient survival, as well as the incidence of malignancies.

## Conclusions

Our study found that lower cumulative doses (2 or 2.5 mg/kg) of rATG performed comparably to higher doses (≥3 mg per kg) in preventing acute rejection and maintaining graft survival in low-risk KTRs. These results suggest that lower rATG doses can reduce the risk of adverse effects while maintaining therapeutic efficacy. However, the choice of dose should be individualized, considering factors such as the patient’s immunological risk, overall health, and the center’s experience. Future research should focus on refining the induction therapy protocols by exploring rATG dosing in high-risk populations. This might guide us in optimizing immunosuppression therapy and thereby enhancing transplant outcomes.

## References

[1] Andress L, Gupta A, Siddiqi N, Marfo K (2014). Rabbit anti-thymocyte globulin induction in renal transplantation: review of the literature. Transpl Res Risk Manage.

[2] Bamoulid J, Staeck O, Crépin T (2017). Anti-thymocyte globulins in kidney transplantation: focus on current indications and long-term immunological side effects. Nephrol Dial Transplant.

[3] Grafals M, Smith B, Murakami N (2014). Immunophenotyping and efficacy of low dose ATG in non-sensitized kidney recipients undergoing early steroid withdrawal: a randomized pilot study. PLoS One.

[4] Klem P, Cooper JE, Weiss AS, Gralla J, Owen P, Chan L, Wiseman AC (2009). Reduced dose rabbit anti-thymocyte globulin induction for prevention of acute rejection in high-risk kidney transplant recipients. Transplantation.

[5] Meier-Kriesche HU, Arndorfer JA, Kaplan B (2002). Association of antibody induction with short- and long-term cause-specific mortality in renal transplant recipients. J Am Soc Nephrol.

[6] Opelz G, Naujokat C, Daniel V, Terness P, Döhler B (2006). Disassociation between risk of graft loss and risk of non-Hodgkin lymphoma with induction agents in renal transplant recipients. Transplantation.

[7] Thibaudin D, Alamartine E, Mariat C, Absi L, Berthoux F (2005). Long-term kinetic of T-lymphocyte subsets in kidney-transplant recipients: influence of anti-T-cell antibodies and association with posttransplant malignancies. Transplantation.

[8] Laftavi MR, Patel S, Soliman MR, Alnimri M, Kohli R, Said M, Pankewycz O (2011). Low-dose thymoglobulin use in elderly renal transplant recipients is safe and effective induction therapy. Transplant Proc.

[9] Mannick JA, Davis RC, Cooperband SR (1971). Clinical use of rabbit antihuman lymphocyte globulin in cadaver-kidney transplantation. N Engl J Med.

[10] Cardella CJ, Cattran D, Fenton SA, Albert S, Robinette M, Cole E (1997). Induction therapy with rabbit antithymocyte sera reduces rejection episodes in immunologically low-risk living donor renal transplant recipients. Transplant Proc.

[11] Szczech LA, Berlin JA, Aradhye S, Grossman RA, Feldman HI (1997). Effect of anti-lymphocyte induction therapy on renal allograft survival: a meta-analysis. J Am Soc Nephrol.

[12] Brennan DC, Flavin K, Lowell JA (1999). A randomized, double-blinded comparison of thymoglobulin versus Atgam for induction immunosuppressive therapy in adult renal transplant recipients. Transplantation.

[13] (2009). KDIGO clinical practice guideline for the care of kidney transplant recipients. Am J Transplant.

[14] Büchler M, Hurault de Ligny B, Madec C, Lebranchu Y (2003). Induction therapy by anti-thymocyte globulin (rabbit) in renal transplantation: a 1-yr follow-up of safety and efficacy. Clin Transplant.

[15] Stevens RB, Mercer DF, Grant WJ (2008). Randomized trial of single-dose versus divided-dose rabbit anti-thymocyte globulin induction in renal transplantation: an interim report. Transplantation.

[16] Wong W, Agrawal N, Pascual M (2006). Comparison of two dosages of thymoglobulin used as a short-course for induction in kidney transplantation. Transpl Int.

[17] Gurk-Turner C, Airee R, Philosophe B, Kukuruga D, Drachenberg C, Haririan A (2008). Thymoglobulin dose optimization for induction therapy in high risk kidney transplant recipients. Transplantation.

[18] Tanriover B, Chuang P, Fishbach B (2005). Polyclonal antibody-induced serum sickness in renal transplant recipients: treatment with therapeutic plasma exchange. Transplantation.

[19] Mueller TF (2007). Mechanism of action of thymoglobulin. Transplantation.

[20] Gaber AO, Matas AJ, Henry ML (2012). Antithymocyte globulin induction in living donor renal transplant recipients: final report of the TAILOR registry. Transplantation.

[21] Laftavi MR, Alnimri M, Weber-Shrikant E, Kohli R, Said M, Patel S, Pankewycz O (2011). Low-dose rabbit antithymocyte globulin versus basiliximab induction therapy in low-risk renal transplant recipients: 8-year follow-up. Transplant Proc.

[22] Sigurjonsdottir VK, Maestretti L, McGrath A (2022). Low dose rabbit antithymocyte globulin is non-inferior to higher dose in low-risk pediatric kidney transplant recipients. Pediatr Nephrol.

[23] Singh N, Rossi AP, Savic M, Rubocki RJ, Parker MG, Vella JP (2018). Tailored rabbit antithymocyte globulin induction dosing for kidney transplantation. Transplant Direct.

[24] Schenker P, Ozturk A, Vonend O (2011). Single-dose thymoglobulin induction in living-donor renal transplantation. Ann Transplant.

[25] Ray D S, Gang S, Khullar D (2023). Rabbit anti-thymocyte globulin (rATG) as induction immunosuppression therapy in patients undergoing renal transplantation: clinical experience from RISE registry. Indian J Transpl.

[26] Noël C, Abramowicz D, Durand D (2009). Daclizumab versus antithymocyte globulin in high-immunological-risk renal transplant recipients. J Am Soc Nephrol.

[27] Thiyagarajan UM, Ponnuswamy A, Bagul A (2013). Thymoglobulin and its use in renal transplantation: a review. Am J Nephrol.

[28] Clesca P, Dirlando M, Park SI (2007). Thymoglobulin and rate of infectious complications after transplantation. Transplant Proc.

[29] Nemati E, Einollahi B, Lesan Pezeshki M, Porfarziani V, Fattahi MR (2014). Does kidney transplantation with deceased or living donor affect graft survival?. Nephrourol Mon.

[30] Kute VB, Vanikar AV, Shah PR (2014). Outcome of live and deceased donor renal transplantation in patients aged ≥55 years: a single-center experience. Indian J Nephrol.

